# Supplementary material for: Expression and processing of mature human frataxin after gene therapy in mice
Source: Sci Rep. 2024 Apr 10;14:8391. doi: 10.1038/s41598-024-59060-0 (PMC11006666; doi:10.1038/s41598-024-59060-0)
Supplement: Supplementary file 1 — Supplementary Information. [file 41598_2024_59060_MOESM1_ESM.pdf]

Rojsajjakul et al.
Supplementary information

Expression and processing of mature human frataxin after gene therapy in mice

Teerapat Rojsajjakul[1], Nithya Selvan[2], Bishnu De[3], Jonathan B. Rosenberg[3], Stephen M. Kaminsky[3], Dolan Sondhi[3], Peter Janki[2], Ronald G. Crystal[3], Clementina Mesaros[1], Richie Khanna[2], and Ian A. Blair[1]

[1]Penn/CHOP Friedreich's Ataxia Center of Excellence, Center of Excellence in Environmental Toxicology, and Department of Systems Pharmacology and Translational Therapeutics Perelman School of Medicine, University of Pennsylvania Philadelphia, PA 19104, United States

[2]LEXEO Therapeutics, Inc, New York, NY 10010, United States

[3]Department of Genetic Medicine, Weill Cornell College of Medicine, New York, NY 10065, United States

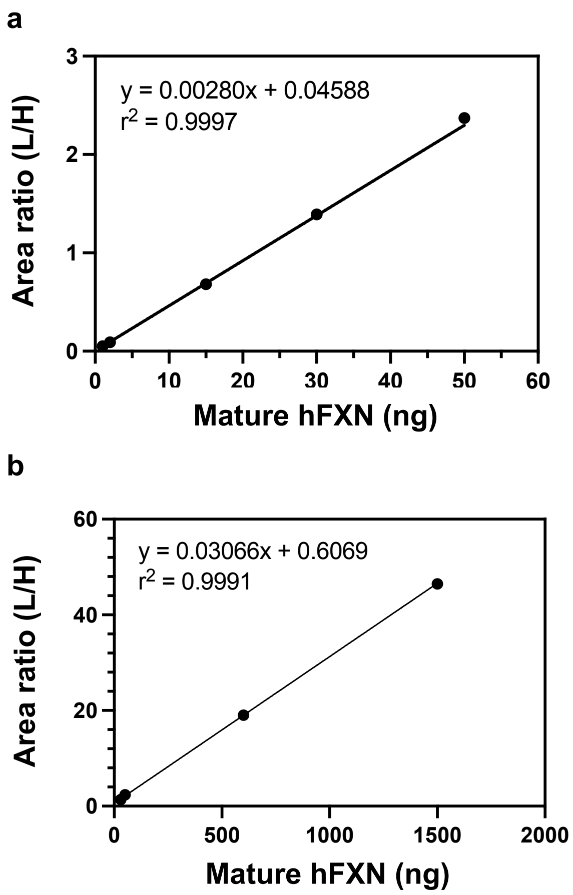

Supplementary Figure S1. Standard curves for analysis of mature hFXN. (**a**) 1–50 ng. (**b**) 50–1500 ng. hFXN = human frataxin; L/H = Light-to-heavy peptides ratio (i.e., ratio of S^{81}GTLGHPGSLDETTYER^{97} to S^{81}GT**L**GHPGS**L**DETTYER^{97}).

a

Start	End	Truncation/ Elongation	Peptide	Human/ Mouse	L/H	Parent ion	Parent ion (m/z)	Product ion	Product ion (m/z)
81	97	N	SGT**L**GHPGS**L**DETTYER	Human	H	MH_3^{3+}	611.300	y_5^+	669.320
77	94	N+1	NLGTLDNPSSLDETAYER	Mouse	L	MH_2^{2+}	997.969	y_7^+	883.379
78	94	N	LGTLDNPSSLDETAYER	Mouse	L	MH_2^{2+}	940.947	y_7^+	883.379
79	94	N-1	GTLDNPSSLDETAYER	Mouse	L	MH_2^{2+}	884.4052	y_7^+	883.379
80	94	N-2	TLDNPSSLDETAYER	Mouse	L	MH_2^{2+}	855.895	y_7^+	883.379
81	94	N-3	LDNPSSLDETAYER	Mouse	L	MH_2^{2+}	805.371	y_7^+	883.379
82	94	N-4	DNPSSLDETAYER	Mouse	L	MH_2^{2+}	748.829	y_7^+	883.379
83	94	N-5	NPSSLDETAYER	Mouse	L	MH_2^{2+}	691.315	y_7^+	883.379
84	94	N-6	PSSLDETAYER	Mouse	L	MH_2^{2+}	634.394	y_7^+	883.379

b

Start	End	Truncation	Peptide	Human/ Mouse	L/H	Parent ion	Parent ion (m/z)	Product ion	Product ion (m/z)
81	97	N	SGT**L**GHPGS**L**DETTYER	Human	H	MH_3^{3+}	611.300	y_5^+	669.320
81	97	N	SGTLGHPGSLDETTYER	Human	L	MH_3^{3+}	607.287	y_5^+	669.320
82	97	N-1	GTLGHPGSLDETTYER	Human	L	MH_3^{3+}	578.276	y_5^+	669.320
83	97	N-2	TLGHPGSLDETTYER	Human	L	MH_3^{3+}	559.269	y_5^+	669.320
84	97	N-3	LGHPGSLDETTYER	Human	L	MH_3^{3+}	525.586	y_5^+	669.320
85	97	N-4	GHPGSLDETTYER	Human	L	MH_3^{3+}	487.892	y_5^+	669.320
86	97	N-5	HPGSLDETTYER	Human	L	MH_3^{3+}	468.885	y_5^+	669.320
87	97	N-6	PGSLDETTYER	Human	L	MH_3^{3+}	634.294	y_5^+	669.320

Supplementary Table S1. PRM/HRMS ions monitored for analysis of FXN proteoforms in mouse heart and liver tissue homogenates. (**a**) mFXN. (**b**) hFXN. FXN = frataxin; hFXN = human frataxin; **L** = [$^{13}C_6$] = Leucine; L/H = Light-to-heavy peptides ratio; mFXN = mouse frataxin; m/z = mass-to-charge ratio; PRM/HRMS = parallel reaction monitoring-mass spectrometry.

a

Theoretical (ng)	Area ratio (L/H)	Slope	Intercept	Calculated (ng)	DEV (%)
1	0.050	0.04588	0.00288	1.03	3
2	0.090	0.04588	0.00288	2.03	2
15	0.675	0.04588	0.00288	14.78	−1
30	1.390	0.04588	0.00288	30.36	1
50	2.368	0.04588	0.00288	51.67	3

b

Theoretical (ng)	Area ratio (L/H)	Slope	Intercept	Calculated (ng)	DEV (%)
30	1.390	0.03066	0.6069	25.54	−15
50	2.368	0.03066	0.6069	57.42	15
600	19.011	0.03066	0.6069	600.26	0
1500	46.490	0.03066	0.6069	1496.53	0

Supplementary Table S2. Back-calculated frataxin amounts from typical standard curves. (**a**) 1–50 ng. (**b**) 50–1500 ng. DEV = deviation; L/H = Light-to-heavy peptides ratio.

Animal ID	Dose	NLG (N+1) ng/mg	LGT (N) ng/mg	GTL (N-1) ng/mg	TLD (N-2) ng/mg	LDN (N-3) ng/mg	DNP (N-4) ng/mg	NPS (N-5) ng/mg	PSS (N-6) ng/mg	Total ng/mg	NLG (N+1) %	LGT (N) %	GTL (N-1) %	TLD (N-2) %	LDN (N-3) %	DNP (N-4) %	NPS (N-5) %	PSSL (N-6) %	Total %
FA-B1-08	Veh	0.0	0.4	0.1	0.6	0.0	0.0	0.6	6.0	7.7	0.1	7.2	71.8	1.6	9.1	0.4	0.3	9.4	100.0
FA-B1-09	Veh	0.0	3.2	13.3	0.4	1.1	0.1	0.1	3.5	21.8	0.1	14.6	61.2	1.8	5.2	0.5	0.4	16.3	100.0
FA-B1-10	Veh	0.0	0.8	4.3	0.1	0.2	0.0	0.0	0.5	6.0	0.1	12.9	71.3	2.4	3.5	0.3	0.4	9.1	100.0
FA-B1-20	Veh	0.2	26.9	112.0	4.8	13.4	0.8	0.7	34.7	193.5	0.1	13.9	57.8	2.5	6.9	0.4	0.4	17.9	100.0
FA-B1-21	Veh	0.1	11.8	49.7	1.3	1.8	0.4	0.3	4.7	70.1	0.1	16.8	70.9	1.9	2.5	0.6	0.4	6.8	100.0
FA-B1-08	**Mean**	**0.1**	**8.6**	**35.9**	**1.4**	**3.3**	**0.3**	**0.3**	**9.9**	**59.8**	**0.1**	**13.1**	**66.6**	**2.0**	**5.5**	**0.4**	**0.4**	**11.9**	**100.0**
FA-A1-10	Low	0.0	0.8	3.3	0.1	0.3	0.0	0.0	0.6	5.2	0.0	15.1	64.3	1.7	6.7	0.3	0.2	11.7	100.0
FA-A1-11	Low	0.0	3.7	19.6	0.7	2.0	0.1	0.3	6.7	33.1	0.1	11.3	59.1	2.1	6.1	0.3	0.8	20.2	100.0
FA-A1-12	Low	0.0	0.9	4.7	0.1	0.2	0.0	0.0	0.6	6.5	0.1	13.6	71.9	1.6	2.7	0.2	0.3	9.7	100.0
FA-A1-23	Low	0.0	0.8	3.3	0.1	0.1	0.0	0.0	0.4	4.7	0.1	17.6	70.6	1.3	2.5	0.1	0.3	7.6	100.0
FA-A1-24	Low	0.0	1.1	5.6	0.1	0.4	0.0	0.0	0.4	7.6	0.1	12.8	74.2	1.6	5.4	0.3	0.3	5.2	100.0
FA-E1-11	Low	0.0	0.8	3.8	0.1	0.5	0.0	0.0	0.1	5.4	0.1	15.3	70.1	2.2	9.4	0.2	0.1	2.6	100.0
FA-A1-10	**Mean**	**0.0**	**1.3**	**6.7**	**0.2**	**0.6**	**0.0**	**0.1**	**1.5**	**10.4**	**0.1**	**14.3**	**68.4**	**1.7**	**5.5**	**0.2**	**0.4**	**9.5**	**100.0**
FA-C1-10	Mid	0.0	1.2	6.6	0.2	0.5	0.0	0.0	1.2	9.8	0.1	12.4	67.6	2.3	4.9	0.4	0.4	11.9	100.0
FA-C1-11	Mid	0.0	2.7	11.6	0.3	1.2	0.0	0.1	1.4	17.3	0.0	15.4	67.3	1.9	7.2	0.0	0.3	7.9	100.0
FA-C1-12	Mid	0.0	7.3	20.4	0.7	0.7	0.1	0.1	3.4	32.7	0.0	22.5	62.5	2.1	2.1	0.2	0.4	10.3	100.0
FA-C1-22	Mid	0.0	1.1	6.2	0.2	0.6	0.0	0.0	0.9	9.0	0.1	12.2	68.5	2.4	6.5	0.4	0.3	9.6	100.0
FA-C1-23	Mid	missing																	
FA-C1-24	Mid	missing																	
FA-C1-10	**Mean**	**0.0**	**3.1**	**11.2**	**0.4**	**0.7**	**0.0**	**0.1**	**1.7**	**17.20**	**0.0**	**15.6**	**66.5**	**2.2**	**5.2**	**0.3**	**0.4**	**9.9**	**100.0**
FA-D1-07	High	0.0	0.6	2.7	0.1	0.1	0.0	0.0	0.3	3.8	0.1	14.6	72.0	2.3	2.4	0.3	0.4	7.9	100.0
FA-D1-10	High	0.1	18.7	56.4	1.7	7.3	0.4	0.4	11.7	96.7	0.1	19.3	58.3	1.8	7.6	0.4	0.5	12.1	100.0
FA-D1-12	High	0.0	2.6	8.1	0.3	0.7	0.1	0.2	2.9	14.8	0.1	17.7	54.4	1.8	4.7	0.6	1.2	19.6	100.0
FA-D1-20	High	0.0	5.7	19.5	0.6	1.0	0.1	0.1	5.1	32.1	0.1	17.9	60.6	1.8	3.0	0.3	0.4	16.0	100.0
FA-D1-21	High	0.0	0.4	2.9	0.1	0.2	0.0	0.0	0.3	3.8	0.1	10.0	76.1	1.6	4.4	0.4	0.3	7.1	100.0
FA-D1-22	High	0.0	0.7	3.9	0.1	0.3	0.0	0.0	0.2	5.2	0.1	13.1	74.1	2.1	5.1	0.4	0.2	4.8	100.0
FA-D1-23	High	0.0	0.2	1.4	0.0	0.2	0.0	0.0	0.1	1.9	0.1	9.9	74.6	2.0	8.4	0.6	0.3	4.2	100.0
FA-D1-24	High	0.0	0.2	1.0	0.0	0.0	0.0	0.0	0.1	1.3	0.1	13.7	74.6	2.9	2.8	0.6	0.4	4.9	100.0
	**Mean**	**0.0**	**3.6**	**12.0**	**0.4**	**1.2**	**0.1**	**0.1**	**2.6**	**19.9**	**0.1**	**14.5**	**68.1**	**2.0**	**4.8**	**0.5**	**0.5**	**11.9**	**100.0**
**Overall Mean**		**0.0**	**4.0**	**15.7**	**0.6**	**1.4**	**0.1**	**0.1**	**3.7**	**25.6**	**0.1**	**14.3**	**67.6**	**2.0**	**5.2**	**0.4**	**0.4**	**10.1**	**100.0**

Supplementary Table S3. Levels and relative amounts of mature mFXN proteoforms in the hearts of mice after intravenously administering AAVrh.10hFXN. While there were six mice treated with mid-dose AAVrh.10hFXN, insufficient heart sample precluded analysis of two mice. AAVrh.10hFXN = adeno-associated virus rhesus serotype 10 encoding human frataxin; mFXN = mouse frataxin.

Animal ID	Dose	NLG (N+1) ng/mg	LGT (N) ng/mg	GTL (N-1) ng/mg	TLD (N-2) ng/mg	LDN (N-3) ng/mg	DNP (N-4) ng/mg	NPS (N-5) ng/mg	PSS (N-6) ng/mg	Total ng/mg	NLG (N+1) %	LGT (N) %	GTL (N-1) %	TLD (N-2) %	LDN (N-3) %	DNP (N-4) %	NPS (N-5) %	PSSL (N-6) %
FA-B1-08	Veh	0.0	4.0	9.8	0.4	3.6	0.0	0.0	1.7	19.6	0.1	20.2	50.1	2.1	18.5	0.0	0.1	8.9
FA-B1-09	Veh	0.0	2.5	6.6	0.5	4.1	0.0	0.0	1.3	14.9	0.1	16.8	43.9	3.1	27.3	0.0	0.2	8.7
FA-B1-10	Veh	0.1	14.3	39.4	1.4	10.7	0.1	0.1	5.7	71.7	0.1	19.9	55.0	1.9	15.0	0.1	0.1	7.9
FA-B1-20	Veh	0.0	1.7	5.2	0.2	4.0	0.0	0.0	1.0	12.0	0.1	14.0	43.1	1.6	33.1	0.1	0.2	8.0
FA-B1-21	Veh	0.0	1.5	7.1	0.1	1.8	0.0	0.0	0.7	11.3	0.0	13.7	63.0	1.3	16.0	0.0	0.0	6.0
	**Mean**	**0.0**	**4.8**	**13.6**	**0.5**	**4.8**	**0.0**	**0.0**	**2.1**	**25.9**	**0.1**	**16.9**	**51.0**	**2.0**	**22.0**	**0.0**	**0.1**	**7.9**
FA-A1-10	Low	0.0	0.5	2.2	0.1	1.5	0.0	0.0	0.3	4.7	0.0	11.0	47.3	1.9	32.6	0.1	0.2	6.9
FA-A1-11	Low	0.0	0.4	2.4	0.1	0.5	0.0	0.0	0.3	3.7	0.1	11.4	64.9	1.5	13.3	0.8	0.8	7.4
FA-A1-12	Low	0.0	0.7	2.6	0.2	2.8	0.0	0.0	0.5	6.8	0.1	10.2	38.5	2.3	40.9	0.0	0.2	7.9
FA-A1-23	Low	0.0	2.3	10.5	0.5	8.4	0.5	0.3	2.4	24.9	0.1	9.1	42.1	1.8	33.8	2.2	1.3	9.7
FA-A1-24	Low	0.0	1.0	3.2	0.5	9.6	0.0	0.1	1.5	15.7	0.0	6.3	20.1	3.0	60.8	0.0	0.5	9.2
FA-E1-11	Low	0.0	4.0	15.0	2.1	51.2	2.6	1.6	5.1	81.7	0.0	4.9	18.4	2.6	62.7	3.1	2.0	6.2
	**Mean**	**0.0**	**1.5**	**6.0**	**0.6**	**12.3**	**0.5**	**0.3**	**1.7**	**22.9**	**0.1**	**8.8**	**38.5**	**2.2**	**40.7**	**1.0**	**0.8**	**7.9**
FA-C1-10	Mid	0.0	0.7	2.6	0.1	1.9	0.0	0.0	0.4	5.8	0.1	11.9	44.7	2.2	33.5	0.0	0.2	7.4
FA-C1-11	Mid	0.0	1.5	5.1	0.2	2.5	0.0	0.0	0.7	10.1	0.1	14.5	50.9	2.4	24.8	0.1	0.1	7.0
FA-C1-12	Mid	0.0	0.7	2.7	0.3	4.5	0.0	0.0	0.7	8.9	0.0	8.2	30.6	3.1	50.3	0.0	0.3	7.4
FA-C1-22	Mid	0.0	0.6	1.8	0.1	2.2	0.0	0.0	0.4	5.2	0.0	12.4	35.2	2.9	42.5	0.0	0.2	6.9
FA-C1-23	Mid	0.0	0.6	1.8	0.2	3.8	0.0	0.0	0.6	7.00	0.1	8.5	26.1	2.8	54.0	0.0	0.3	8.1
FA-C1-24	Mid	0.0	0.8	3.6	0.2	4.6	0.0	0.0	0.6	9.9	0.0	8.5	36.7	2.4	46.3	0.0	0.2	6.0
	**Mean**	**0.0**	**0.8**	**2.9**	**0.2**	**3.2**	**0.0**	**0.0**	**0.6**	**8.2**	**0.0**	**10.7**	**37.4**	**2.6**	**41.9**	**0.0**	**0.2**	**7.2**
FA-D1-07	High	0.0	0.8	4.1	0.4	3.3	0.4	0.2	1.1	10.2	0.1	8.3	40.4	3.7	31.9	3.5	1.8	10.3
FA-D1-10	High	0.0	0.8	2.7	0.1	2.2	0.0	0.0	0.5	6.2	0.0	12.3	43.1	2.0	34.9	0.0	0.2	7.4
FA-D1-12	High	0.0	0.3	1.1	0.1	1.0	0.5	0.0	0.2	3.4	0.1	9.7	33.3	2.6	30.7	15.4	1.1	7.0
FA-D1-20	High	0.0	0.8	3.4	0.2	4.3	0.0	0.0	0.6	9.5	0.0	8.5	36.4	2.3	45.9	0.0	0.1	6.8
FA-D1-21	High	0.0	1.0	3.5	0.3	4.8	0.2	0.1	0.9	10.9	0.1	8.9	32.1	2.9	44.6	2.2	1.2	8.0
FA-D1-22	High	0.0	0.8	2.5	0.2	2.9	0.2	0.1	0.7	7.4	0.1	10.5	34.1	3.0	38.8	2.4	1.3	9.9
FA-D1-23	High	0.0	0.3	1.2	0.1	0.8	0.2	0.0	0.2	2.7	0.0	12.1	44.7	2.1	30.4	2.3	1.2	7.2
FA-D1-24	High	0.0	0.8	3.6	0.2	2.5	0.2	0.0	0.6	7.9	0.1	10.2	45.4	2.8	31.2	1.9	0.6	7.9
	**Mean**	**0.0**	**0.7**	**2.8**	**0.2**	**2.7**	**0.2**	**0.1**	**0.6**	**7.7**	**0.1**	**10.0**	**38.7**	**2.7**	**36.0**	**3.5**	**0.9**	**8.1**
**Overall mean**		**0.0**	**1.7**	**5.8**	**0.4**	**5.6**	**0.2**	**0.1**	**1.1**	**14.9**	**0.1**	**11.3**	**40.8**	**2.4**	**35.8**	**1.4**	**0.6**	**7.8**

Supplementary Table S4. Levels and relative amounts of mature mFXN proteoforms in the livers of mice after intravenously administering AAVrh.10hFXN. AAVrh.10hFXN = adeno-associated virus rhesus serotype 10 encoding human frataxin; mFXN = mouse frataxin.

Animal ID	Dose	SGT (N) ng/mg	GTL (N-1) ng/mg	TLG (N-2) ng/mg	LGH (N-3) ng/mg	GHP (N-4) ng/mg	HPG (N-5) ng/mg	PGS (N-6) ng/mg	Total ng/mg	SGT (N) %	GTL (N-1) %	TLG (N-2) %	LGH (N-3) %	GHP (N-4) %	HPG (N-5) %	PGS (N-6) %	Total %
FA-B1-08	Veh	0.1	0.0	0.0	0.0	0.0	0.0	0.0	0.2	67.9	11.9	5.3	0.0	0.0	0.0	14.9	100.0
FA-B1-09	Veh	0.0	0.0	0.0	0.0	0.0	0.0	0.0	0.0	65.1	2.3	8.2	3.3	18.3	2.9	0.0	100.0
FA-B1-10	Veh	1.1	0.1	0.1	0.0	0.0	0.0	0.4	1.6	66.1	7.3	3.8	0.0	0.3	0.0	22.6	100.0
FA-B1-20	Veh	0.0	0.0	0.0	0.0	0.0	0.0	0.0	0.0	96.3	1.7	2.0	0.0	0.0	0.0	0.0	100.0
FA-B1-21	Veh	0.7	0.0	0.0	0.0	0.0	0.0	0.0	0.7	92.6	1.4	6.1	0.0	0.0	0.0	0.0	100.0
	**Mean**	**0.4**	**0.0**	**0.0**	**0.0**	**0.0**	**0.0**	**0.1**	**0.5**	**77.6**	**4.9**	**5.1**	**0.7**	**3.7**	**0.6**	**7.5**	**100.0**
FA-A1-10	Low	0.1	0.0	0.0	0.0	0.0	0.0	0.0	0.2	81.4	0.1	0.0	2.0	7.0	0.0	9.5	100.0
FA-A1-11	Low	0.3	0.0	0.0	0.0	0.0	0.0	0.0	0.3	76.2	3.0	9.0	0.0	9.8	1.8	0.1	100.0
FA-A1-12	Low	0.1	0.0	0.0	0.0	0.0	0.0	0.0	0.1	89.6	0.0	0.0	0.0	0.0	0.0	10.4	100.0
FA-A1-23	Low	0.1	0.0	0.0	0.0	0.0	0.0	0.0	0.1	86.8	3.1	3.0	0.0	0.0	0.0	7.1	100.0
FA-A1-24	Low	0.0	0.0	0.0	0.0	0.0	0.0	0.0	0.1	75.3	8.0	6.3	0.3	0.0	0.0	10.2	100.0
FA-E1-11	Low	0.2	0.0	0.0	0.0	0.0	0.0	0.0	0.2	83.6	0.3	10.9	1.4	1.7	0.0	2.0	100.0
	**Mean**	**0.1**	**0.0**	**0.0**	**0.0**	**0.0**	**0.0**	**0.0**	0.2	**82.1**	**2.4**	**4.9**	**0.6**	**3.1**	**0.3**	**6.6**	**100.0**
FA-C1-10	Mid	0.4	0.0	0.0	0.0	0.0	0.0	0.0	0.5	86.5	3.1	8.3	0.2	1.0	0.1	0.9	100.0
FA-C1-11	Mid	0.9	0.0	0.0	0.0	0.0	0.0	0.0	1.0	97.0	0.3	0.0	0.0	2.5	0.0	0.1	100.0
FA-C1-12	Mid	5.5	0.3	0.7	0.0	0.1	0.0	0.0	6.7	83.1	4.0	10.7	0.5	1.1	0.5	0.1	100.0
FA-C1-22	Mid	13.9	0.3	1.9	0.0	0.1	0.0	0.0	16.2	85.9	1.6	11.6	0.1	0.8	0.0	0.0	100.0
FA-C1-23	Mid	missing															
FA-C1-24	Mid	missing															
	**Mean**	**5.2**	**0.1**	**0.7**	**0.0**	**0.1**	**0.0**	**0.0**	**6.1**	**88.1**	**2.3**	**7.6**	**0.2**	**1.3**	**0.1**	**0.3**	**100.0**
FA-D1-07	High	5.2	0.3	0.0	0.0	0.0	0.0	0.0	5.6	93.8	4.7	0.2	0.2	0.5	0.6	0.1	100.0
FA-D1-10	High	10.6	0.2	0.8	0.0	0.1	0.2	0.0	12.0	88.8	1.7	6.4	0.4	1.1	1.3	0.2	100.0
FA-D1-12	High	46.9	1.7	4.7	0.4	1.0	1.0	0.1	55.8	84.1	3.1	8.5	0.6	1.7	1.8	0.1	100.0
FA-D1-20	High	50.2	1.9	2.7	0.1	0.6	0.4	0.1	56.0	89.6	3.3	4.9	0.3	1.1	0.6	0.1	100.0
FA-D1-21	High	40.7	1.2	4.0	0.1	0.0	0.0	0.1	46.2	88.2	2.7	8.6	0.3	0.1	0.0	0.2	100.0
FA-D1-22	High	9.8	0.2	0.6	0.0	0.0	0.0	0.0	10.8	91.4	2.2	5.5	0.2	0.3	0.1	0.3	100.0
FA-D1-23	High	8.2	0.3	0.9	0.0	0.0	0.0	0.0	9.5	86.9	3.1	9.2	0.5	0.0	0.1	0.3	100.0
FA-D1-24	High	3.7	0.1	0.2	0.0	0.0	0.0	0.0	4.0	90.8	2.5	5.7	0.3	0.2	0.1	0.4	100.0
	**Mean**	**21.9**	**0.7**	**1.7**	**0.1**	**0.2**	**0.2**	**0.0**	**25.0**	**89.2**	**2.9**	**6.1**	**0.3**	**0.6**	**0.6**	**0.2**	**100.0**
**Overall Mean**		**8.7**	**0.3**	**0.7**	**0.0**	**0.1**	**0.1**	**0.0**	**9.9**	**84.6**	**3.1**	**5.8**	**0.5**	**2.1**	**0.4**	**3.5**	**100.0**

Supplementary Table S5. Levels and relative amounts of mature hFXN proteoforms in the hearts of mice after intravenously administering AAVrh.10hFXN. While there were six mice treated with mid-dose AAVrh.10hFXN, insufficient heart sample precluded analysis of two mice. AAVrh.10hFXN = adeno-associated virus rhesus serotype 10 encoding human frataxin; hFXN = human frataxin.

Animal ID	Dose	SGT (N) ng/mg	GTL (N-1) ng/mg	TLG (N-2) ng/mg	LGH (N-3) ng/mg	GHP (N-4) ng/mg	HPG (N-5) ng/mg	PGS (N-6) ng/mg	Total ng/mg	SGT (N) %	GTL (N-1) %	TLG (N-2) %	LGH (N-3) %	GHP (N-4) %	HPG (N-5) %	PGS (N-6) %	Total %
FA-B1-08	Veh	0.2	0.0	0.1	0.0	0.1	0.0	0.0	0.4	43.2	2.4	14.4	3.7	32.0	3.4	1.0	100.0
FA-B1-09	Veh	0.1	0.0	0.0	0.0	0.1	0.0	0.0	0.3	52.2	3.9	15.4	5.3	20.5	2.6	0.1	100.0
FA-B1-10	Veh	4.5	0.3	1.3	0.3	1.6	0.0	0.2	8.2	55.2	3.3	16.1	3.4	20.1	0.0	1.9	100.0
FA-B1-20	Veh	0.0	0.0	0.0	0.0	0.0	0.0	0.0	0.0	54.1	2.5	12.8	4.7	23.9	0.0	2.0	100.0
FA-B1-21	Veh	14.0	0.0	3.8	0.0	13.3	0.0	0.7	31.8	43.9	0.0	12.1	0.0	41.8	0.0	2.2	100.0
	**Mean**	**3.8**	**0.1**	**1.1**	**0.1**	**3.0**	**0.0**	**0.2**	**8.1**	**49.7**	**2.4**	**14.2**	**3.4**	**27.7**	**1.2**	**1.4**	**100.0**
FA-A1-10	Low	19.7	1.0	5.6	1.3	6.9	1.0	0.0	35.6	55.3	2.9	15.8	3.7	19.5	2.7	0.1	100.0
FA-A1-11	Low	20.9	0.9	2.6	0.6	1.6	0.4	0.0	27.0	77.4	3.2	9.8	2.1	5.8	1.6	0.1	100.0
FA-A1-12	Low	19.3	0.8	7.5	1.1	9.8	0.8	0.0	39.3	49.0	2.1	19.0	2.9	25.0	2.0	0.1	100.0
FA-A1-23	Low	11.4	0.6	3.0	1.2	4.3	0.5	0.0	21.1	54.2	2.7	14.3	5.7	20.4	2.5	0.1	100.0
FA-A1-24	Low	6.9	0.4	2.5	0.9	12.6	1.5	0.0	24.9	27.9	1.6	9.9	3.8	50.7	6.1	0.1	100.0
FA-E1-11	Low	0.2	0.0	0.0	0.0	0.3	0.0	0.0	0.5	29.6	1.5	8.1	3.1	50.0	7.6	0.1	100.0
	**Mean**	**13.1**	**0.6**	**3.5**	**0.9**	**5.9**	**0.7**	**0.0**	**24.7**	**48.9**	**2.3**	**12.8**	**3.5**	**28.6**	**3.7**	**0.1**	**100.0**
FA-C1-10	Mid	17.9	1.3	6.4	1.6	5.7	0.7	0.0	33.8	53.1	3.8	19.1	4.7	17.0	2.2	0.1	100.0
FA-C1-11	Mid	10.9	1.0	2.8	0.9	2.1	0.5	0.0	18.2	59.9	5.4	15.6	4.8	11.4	2.8	0.1	100.0
FA-C1-12	Mid	10.1	0.9	3.8	1.7	9.8	1.7	0.0	28.0	36.1	3.2	13.4	6.2	34.8	6.1	0.1	100.0
FA-C1-22	Mid	16.2	2.0	8.2	3.4	10.7	0.0	0.0	40.4	39.9	4.9	20.2	8.3	26.5	0.1	0.1	100.0
FA-C1-23	Mid	8.2	0.4	2.3	1.3	5.8	1.2	0.0	19.3	42.4	2.3	11.8	6.9	30.0	6.4	0.1	100.0
FA-C1-24	Mid	17.2	1.5	6.9	2.5	10.0	0.0	0.01	38.1	45.0	3.9	18.1	6.5	26.2	0.1	0.1	100.0
	**Mean**	**13.4**	**1.2**	**5.1**	**1.9**	**7.3**	**0.7**	**0.0**	**29.6**	**46.1**	**3.9**	**16.4**	**6.3**	**24.3**	**3.0**	**0.1**	**100.0**
FA-D1-07	High	10.1	1.5	4.6	1.9	5.4	1.9	0.0	25.3	39.7	5.8	18.2	7.6	21.3	7.4	0.1	100.0
FA-D1-10	High	16.8	0.6	7.0	0.7	10.5	0.6	0.0	36.4	46.3	1.8	19.3	1.9	29.0	1.7	0.1	100.0
FA-D1-12	High	9.9	0.6	2.2	0.7	3.9	1.2	0.0	18.6	53.1	3.3	12.1	3.9	21.2	6.3	0.1	100.0
FA-D1-20	High	13.4	0.9	5.8	1.1	7.7	0.6	0.0	29.5	45.5	2.9	19.8	3.8	26.0	1.9	0.1	100.0
FA-D1-21	High	14.1	1.3	6.8	2.4	10.6	2.2	0.0	37.5	37.6	3.4	18.2	6.4	28.3	5.9	0.1	100.0
FA-D1-22	High	19.3	2.0	6.4	4.2	14.2	2.7	0.1	49.0	39.5	4.0	13.1	8.6	29.0	5.6	0.1	100.0
FA-D1-23	High	20.9	0.8	6.8	1.0	7.6	0.9	0.0	38.1	54.9	2.0	18.0	2.7	19.9	2.4	0.1	100.0
FA-D1-24	High	6.2	0.3	2.4	0.9	4.5	0.7	0.0	15.2	41.0	2.2	16.1	6.0	29.8	4.7	0.1	100.0
	**Mean**	**13.8**	**1.0**	**5.3**	**1.6**	**8.1**	**1.3**	**0.0**	**31.2**	**44.7**	**3.2**	**16.8**	**5.1**	**25.5**	**4.5**	**0.1**	**100.0**
**Overall Mean**		**11.5**	**0.8**	**4.0**	**1.2**	**6.4**	**0.8**	**0.1**	**24.7**	**47.0**	**3.0**	**15.2**	**4.7**	**26.4**	**3.3**	**0.4**	**100.0**

Supplementary Table S6. Levels and relative amounts of mature hFXN proteoforms in the livers of mice after intravenously administering AAVrh.10hFXN. AAVrh.10hFXN = adeno-associated virus rhesus serotype 10 encoding human frataxin; hFXN = human frataxin.
